# Letheon

**Published:** 1847-06

**Authors:** 


### ARTICLE IX.

### LETHEON.

A spirited war has been, for some time past, carried on between those claiming priority in the discovery of the narcotic effects of the ethereal vapor.

Whether the different claimants will be able to throw any new light upon the subject of its discovery or not does not yet fully appear.

The hottest of the conflict is between Drs. Jackson, Morton, and Wells.

We would respectfully suggest that the patent taken out for its use be honorably relinquished, as it appears the object of it was to secure the title to the discovery, which will now have to be sustained by other evidence. Pray let the profession and the public enjoy the benefits of the application of ether without paying to sustain any one's claim to priority of discovery.

Suppose a patient takes the ether for the insensibility it produces to pain, and, while under its influence, the surgeon performs an operation, charging no other fee than he would be entitled to under other circumstances, what damage would be to pay the patentee? Would damages lie against the surgeon, who has enjoyed none of the advantages of the infringement? or would the patient be subjected to damage for the exemption from pain he has enjoyed?

These are queries that naturally arise in connection with the subject, which *may* be settled by the proper authorities if folks do not quit claiming the discovery of the letheon.

